# The British Journals: Anatomy and Physiology

**Published:** 1839-01

**Authors:** 


### III. THE BRITISH JOURNALS.

(FOR THE QUARTER ENDING NOVEMBER 30, 1838.)

### ANATOMY AND PHYSIOLOGY.

### *On the Mechanism of Bruit de Soufflet, &c.* By D. J. Corrigan, M.D., Physician to Jervis-street Hospital, Dublin.

[In a former Number (Vol. III. p. 257,) we gave an account of Dr. Corrigan's very interesting researches on this subject. The object of the present paper is to corroborate his previous conclusions as to the cause of this phenomenon, and to examine the validity of the opinions advanced by Drs. Williams and Todd, in their Report on the Sounds of the Heart, which we published in our tenth Number. The following brief abstract contains the views of both parties.]

The question at issue between the London committee and me is not a mere abstract question, but one of very direct practical bearing; for it is obvious that, if their report and theory were received as correct, the student and the practitioner would be always led to associate the presence of *bruit de soufflet* with increased resistance, or impediment, or obstruction; and a line of practice might be adopted the very worst adapted for many of those cases in which the sound is heard, and in which the indication very often is, not to lessen force or strength, but to maintain and increase both. The question at issue between us is now within a narrow circle. The experiments and observations are admitted on both sides. Their conclusion is, that "a certain resistance or impediment to a liquid current is the essential physical cause of all murmurs produced by the motions of fluids in elastic tubes. That any condition of the walls of the tube beyond the obstructing point is not, as it has been supposed, essential to the production of these sounds, &c."

My conclusions are, that the conditions necessary to the production of sound are, "1st. A current-like motion of the blood (instead of its natural equable movement,) tending to produce corresponding vibrations in the sides of the cavities or arteries through which it is moving; and, 2dly, a diminished tension of the parietes of the arteries or cavities themselves, in consequence of which these parietes are easily thrown into vibration by the irregular current of the contained fluid; which vibrations cause on the sense of touch, 'fremissement,' and on the sense of hearing, 'bruit de soufflet.'"

The reader can, therefore, now judge for himself of the accuracy of our respective conclusions. *Dublin Journal. November*, 1838.

---

### *On the Structure of the Teeth, the Vascularity of those Organs, and their Relation to Bone.* By John Tomes, Esq.

The microscopical examinations which the author has made of the structure of the teeth of man and various animals lead him to the conclusion that their bony portions are formed of minute tubes, disposed in a radiated arrangement, in lines proceeding everywhere perpendicularly from the inner surface of the cavity containing the pulp. These tubuli are surrounded by a transparent material, which cements them together into a solid and dense mass. He finds, by applying the test of muriatic acid, that carbonate as well as phosphate of lime enters into their composition. In man, the tubuli, during their divergence from their origin at the surface of the central cavity, send off a number of very minute fibrils; and on approaching the enamel or the granular substance, which cover respectively the crown and the fangs of the tooth, the tubuli divide into smaller ones, which freely anastomose with one another, and then either are continued into the enamel, or terminate at the boundary between these two substances. Various modifications of this structure, exhibited in the teeth of different animals, in the class mammalia and fishes more particularly, are minutely described. The granular substance appears to be composed of irregularly shaped osseous granules, imbedded in the same kind of transparent medium which cements the tubuli together. External to the granular portion, the author finds another substance entering into the formation of the simple tooth, and commencing where the enamel terminates; and which he describes as beginning by a thin and transparent layer containing only a few dark fibres, which pass directly outwards; but assuming, as it proceeds towards the apex of the fang, greater thickness and opacity, and being traversed by vessels.

External to the enamel, and in close connexion with it, in compound teeth, is situated the crusta petrosa, a substance very similar to the bony layer of the simple tooth. It contains numerous corpuscles, and is traversed by numerous vessels entering it from without, and anastomosing freely with one another, but terminating in its substance. These investigations of the structure of the different component parts of teeth furnish abundant evidence of their vascularity and consequent vitality. *Proceedings of the Royal Society.* 1837-8. No. 34.

---

### *On the Decussation of Fibres at the junction of the Medulla Spinalis with the Medulla Oblongata.* By John Hilton, Esq.

The author first alludes to what usually happens in affections of the brain, namely, that the loss of voluntary power and of sensation manifest themselves in the opposite side of the body to that in which the cerebral lesion exists, a fact which has been attempted to be explained by the crossing of the fibres at the junction of the medulla oblongata with the anterior or motor columns of the medulla spinalis; but such a structure, he observes, affords no explanation of the loss of sensation. The author then, referring to the communication of Sir Charles Bell to the Royal Society, in the year 1835, describing a decussation connected with the posterior columns, or columns of sensation, mentions that the accuracy of these dissections was doubted by Mr. Mayo and other eminent anatomists. The author proceeds to state that the symptoms of cerebral lesion do not always take place on the opposite side of the body to that in which the lesion of the brain exists, but that they occur sometimes on the same side; that the loss of power and of sensation, although confined to the same side, may exist in either the upper or the lower extremity; but that both are not necessarily implicated; and that, in fact, cases occur where there are marked deviations from what may be considered the more common occurrence. Having observed such cases, and not being aware of any satisfactory explanation, the author examined with care the continuation upwards of the anterior and posterior columns of the spinal marrow into the medulla oblongata and found that the decussation at the upper part of the spinal marrow belonged in part to the columns for motion, and in part to the columns for sensation; and further, that the decussation is only partial with respect to either of these columns; thus elucidating by the observation of the actual structure what before appeared very unsatisfactory in pathology, and anomalous in disease.

The paper is illustrated by drawings made from the dissections of the author.

*Proceedings of the Royal Society.* 1837-8. No. 34.

*On the Causes, Nature, and Prevention of Small-pox.*
By FRANCIS EAGLE, Esq. Surgeon, London.

[THIS paper contains some interesting facts, but more matters of hypothesis and inferences of doubtful authority. The subject, however, at this moment so much engages the attention of the profession that we must find room for the *conclusions* which he draws from the facts and reasonings contained in his essay. It is but justice to Mr. Eagle to state that he regards these conclusions not as all positive, but "subject to the test of experience and further facts."]

First, and principally, that the opinion that small-pox has had but one individual and solitary origin, whence all mankind became infected, is most illogical; that it is founded on our ignorance, and not on facts; that it is negative and not positive; that it is opposed by daily experience of the phenomena of this disease, its rise, its progress and decline, its frequent occurrence where no contagion can possibly be traced, and its deviation in a direct ratio with the causes which produce it; that it is also opposed by the foregoing evidence of its identity, in causes and nature, with cow-pox, which latter disease originates *de novo* frequently, &c.

Secondly. That cow-pox and small-pox are identical:—1. In their causes. 2. In their nature (subject to the law of physical condition). 3. In their effects.

Thirdly. That the circumstance of one being a local the other a general exanthematous affection, is the result of the different anatomical construction of the two animals in which they originate, according to the law which governs the actions of all specific poisons.

Fourthly. That cow-pox, fresh from the cow, is more efficacious than vaccination as now employed.

Fifthly. That the preservative power is temporary and not permanent, preserving the constitution only up to the period of puberty.

Sixthly. That cow-pox, fresh from the cow, and natural small-pox, exercise identical preservative influence over the occurrence of secondary small-pox.

Sevnethly. That the two grand epochs for vaccination are childhood and puberty.

Eighthly. That the inference that the vaccine matter has weakened by time is by no means a necessary or even a fair logical induction, since there is considerable foundation for the opinion that, as all other epidemics are constantly altering their type, deviating in a direct ratio with their causes, the small-pox also does the same.

Ninthly. That the method of reproducing cow-pox consists in bringing the animal under the influence of the same circumstances which are peculiar to the locality of its origin—the dairy counties; namely the early separation of its young.

*Lancet.* October 27, 1838.

---

*New Casts for Anatomical Specimens.* By W. WHITEHOUSE.

[Mr. WHITEHOUSE received the silver medal of the Society of Arts for this mode of casting, which he describes as follows:]

The preparation (whether in the recent state, or having been preserved in spirit or in solution of any sort) is wiped nearly dry, and then arranged, so as best to display its peculiarities, upon a wooden slab of the required size and shape, this having been previously saturated with water, and wiped nearly dry.

Having been slightly fastened to this slab by means of pins, and all those parts in the subsequent relieving of which any difficulty is to be apprehended having been supported or filled up with moistened linen rag, the whole is immersed slowly and gradually (at an angle of forty to forty-five degrees) in the melted composition. This consists of Burgundy pitch and pure bees' wax, each one pound; yellow resin, two oz.; mutton-suet melted and strained, six oz. This should be kept in a fluid state, and at a regular temperature, by means of a lamp placed under the containing vessel. Being withdrawn for a few seconds, it is again and again dipped so as to produce a mould of suitable thickness. This being effected, it is plunged into cold water, when the mould becoming hard, the pins may be easily withdrawn, and the slab and preparation readily taken out of the mould.

The mould is then to be carefully filled with the finest plaster of Paris in the usual way, and in half an hour after this has set, it may be placed for a few minutes in water a little below blood-heat; by this means the mould becomes soft and pliable, and may be easily torn off, bit by bit, in such a manner as to leave uninjured the most delicate part.

Immediately after the removal of the mould, the cast should receive two or three coats of the finest olive or almond oil; when this has been absorbed, we may begin at once to colour from nature, using at the same time a small quantity of fresh ox-gall. *Medical Gazette. September* 29, 1838.

---

*An Account of some Experiments on the Blood in connexion with the Theory of Respiration.* By JOHN DAVY, M.D., F.R.S., Assistant Inspector of Army Hospitals.

The author has investigated, experimentally, several of the important questions connected with the theory of respiration and of animal heat; and arrives at the following results. He finds that the blood is capable of absorbing oxygen both from atmospheric air, and from oxygen gas, independently of putrefaction. After blood has been agitated in common air, a trace of carbonic acid, not exceeding one per cent., is found in the residual air; but when pure oxygen is employed, no carbonic acid can be detected in it by the most carefully conducted trials. When pure carbonic acid is brought into contact with blood, or serum, over mercury, and moderately agitated, the absorption of gas exceeds the volume of the fluid. Both arterial and venous blood are rendered very dark, and serum more liquid by the absorption of this gas to saturation. Serum, in its healthy state, is incapable of absorbing oxygen, or of immediately furnishing carbon to form carbonic acid: and after it has absorbed carbonic acid, only one tenth of the absorbed gas is expelled by successive agitation with atmospheric air, or with hydrogen. The author is inclined to think that the alkali in the blood, in its healthiest condition, is in the state of a sesquicarbonate. In the majority of trials manifest indications of the disengagement of air from blood *in vacuo* were obtained: but as it occasionally happened that no air could be thus extricated, the author is induced to believe that the quantity of air contained in the blood is variable; and he has found this air to consist solely of carbonic acid gas. It would also appear, from the experiments detailed in this paper, that a portion of oxygen exists in the blood, not capable of being extracted by the air-pump, yet capable of entering into combination with nitrous gas; and existing in largest proportion in arterial blood. The absorption of oxygen by blood is attended with an increase of temperature.

The experiments of the author tend to show that the lungs are absorbing and secreting, and perhaps also inhaling organs, and that their peculiar function is to introduce oxygen into the blood, and separate carbonic acid from the blood; and they favour the idea that animal heat is owing, first, to the fixation or condensation of oxygen in the blood in the lungs during its conversion from venous to arterial; and secondly, to the combinations into which it enters in the circulation in connexion with the different secretions and changes essential to animal life.

*Proceedings of the Royal Society.* 1837-8. No. 34.

---

*On some remarkable and hitherto unobserved Phenomena of Binocular Vision.* By CHARLES WHEATSTONE, Esq., F.R.S., Professor of Experimental Philosophy in King's College, London.

THE author first shows that the perspective projections of an object upon the two retinæ differ according to the distance at which the object is placed before the eyes; if it be placed so distant that to view it the optic axes must be parallel, the two projections are precisely similar; but if it be placed so near that to regard it the optic axes must converge, a different perspective projection is presented to each eye; and these perspectives become more dissimilar as the convergence of the optic axis becomes greater. Notwithstanding this dissimilarity between the two pictures, which is in some cases very great, the object is still seen single; contrary to the very prevalent metaphysical opinion, that the single appearance of objects seen by both eyes is owing to their pictures falling on corresponding points of the two retinæ. After establishing these principles, the author proceeds to ascertain what would result from presenting the two monocular perspectives, drawn on plane surfaces, to the two eyes, so that they shall fall on the same parts of the two retinæ as the projections from the object itself would have fallen. Several means are described by which this may be accomplished; but the author especially recommends for this purpose an apparatus called by him a stereoscope, which enables the observer to view the resulting appearances without altering the ordinary adaptation of the eyes, and therefore without subjecting these organs to any strain or fatigue. It consists of two plane mirrors with their backs inclined to each other at an angle of 90°, near the faces of which the two monocular pictures are so placed that their reflected images are seen by the two eyes, one placed before each mirror, in the same place; the apparatus has various adjustments by means of which the magnitude of the images on the retina may be varied, and the optic axis differently converged. If the two monocular pictures be thus presented one to each eye, the mind will perceive, from their combined effect, a figure of three dimensions, the exact counterpart of the object from which the pictures were drawn; to show that this curious illusion does not in the least depend on shading or colouring, the illustrations principally employed are simple outline figures, which give for their perceived resultants skeleton forms of three dimensions. Each monocular outline figure is the representation of two dissimilar skeleton forms, one being the form which it is intended to represent, and another, which Professor Wheatstone calls its converse figure. Viewed by one eye alone the outline may with equal ease be imagined to be either; but when the two monocular pictures are viewed one by each eye, the proper or the complemental form may be fixed in the mind; the former, if the right and left pictures be presented respectively to the right and left eyes; and the latter, if the right picture be presented to the left eye, and the left picture to the right eye. Many new experiments are then detailed, and a variety of instances of false perception of visual objects, some new, others formerly observed, are traced to these principles; among others, the well-known apparent conversion of cameos into intaglios. The author next proceeds to show that pictures similar in form but differing in magnitude within certain limits, when presented one to each eye, are perceived by the mind to be single and of intermediate size; and also that when totally dissimilar pictures, which cannot be combined by the mind into the resemblance of any accustomed objects, are presented one to each eye, they are in general not seen together, but alternately. The memoir concludes with a review of the various hypotheses which have been advanced to account for our seeing objects single with two eyes; and the author states his views respecting the influence which these newly-developed facts are calculated to have on the decision of this much debated question.

*Proceedings of the Royal Society.* 1837-8. No. 34.

---

*Cases of Muscæ Volitantes; with Remarks on their Proximate Cause.*
By W. C. Wallace, m.d., Oculist, New York.

[This is an ingenious and well-written paper, evidently the production of a man of science. Our limits only permit us to extract one of the cases and that portion of the remarks which contain Dr. Wallace's own views.]

Case. A very intelligent gentleman consulted me some time ago about a net-work which appeared before his eyes, and impeded vision. While describing his complaint he drew with his pencil a representation of part of the vascular coat of the retina, as perfectly as if he had had a preparation of the membrane before him for a copy. I took an unusual interest in the case from the drawing, and obtained the following account of it:

"The appearances before my eyes are drawn as correctly as I am able; they do not appear stationary, for when I suddenly throw my eyes up they will also go up, and appear to rise above the object upon which I fix my sight; they then move slowly downward, and sink below the sight, sometimes a little on one side, and at other times exactly in the way, so as to cover a letter or figure at which I may happen to look. I can still see the object, though imperfectly, as if through thin gauze or something of the kind, which dims the sight a little. When they get on one side of the sight, and I attempt to turn my eyes to get a more perfect view of them, turning my eyes appears to turn them also, and they keep at about the same distance. When my eyes are open they do not appear so large nor so plainly as they do when I partly close my eyes. When I fix my sight upon an object, and look steadily at it for a moment or two, without moving my eyes, they will disappear; but the least motion of the eyes will bring them back again as plainly as ever. I have two or three different times noticed a kind of motion, as if caused by hundreds of small insects darting to and fro, when I looked up in the air, in strong day-light. The distance of the net-work appearance seems to be regulated by the distance of the objects at which I look. If I look at an object half a mile off they seem considerably farther from me than they do when I look at an object which is only two or three feet distant. The appearances are worse after a hearty dinner or loss of sleep, and they trouble me least when I feel otherwise well."

When the convex surface of the retina is exposed under water, and scratched with a scalpel, a membrane of great delicacy may be separated, and turned over with the assistance of a camel's hair pencil. This is the coat of Jacob. When the same preparation is allowed to putrefy, and the nervous matter washed away with a camel's hair pencil, the vascular membrane may be exhibited. The ramifications of the blood-vessels in this membrane resemble those of the veins of a leaf after the soft part has been eaten away by insects, and by their intertexture they form a semi-opaque screen, on which is received the image of external objects; just as the ground of a camera obscura, or the screen of a magic lantern.

The nervous matter may be divided into two layers. By allowing an eye to macerate in alcohol, for the purpose of preventing the retina from collapsing when the anterior half of the eye is cut off, and pouring upon the retina thus exposed a watery solution of corrosive sublimate, the fibres may be seen lying beneath the vascular membrane, when they are separated from each other by a camel's hair pencil. In young animals, especially in the calf, the fibres are more easily exhibited than in those which are old. In the human eye, some of them converge round the central foramen. By pouring upon an eye exposed in the same manner an alcoholic solution of corrosive sublimate and muriate of ammonia, the fibrous coat becomes so compact and hard that it may be torn off with forceps, and a layer of globules will be brought into view. These globules are kept in place by the coat of Jacob already described.

The retina, then, consists of four layers—a vascular, a fibrous, a globular, and a serous.

By this exposition of the retina we may account for the various appearances of muscæ volitantes. I have occasionally, when entering an ordinarily lighted room, after a full meal, and exposure to a bright light, witnessed glimmerings like a net-work, which, from its resemblance to the vascular coat, left no doubt in my mind that the blood-vessels of the retina were visible. At other times, in the same circumstances, there was a twisted tube, or a chain of beads, as if there had been an error loci of one of the carved fibres of the retina; or there was a cloud of globules, sometimes packed together, but more frequently separated, and floating in all directions. Each globule was visible for a considerable time, and repeatedly re-occupied the same space. When clustered together, they had a great resemblance to the globules of the retina.

From the similarity of the drawing of the floating network, in Case 1, to the vascular coat of the retina, I am persuaded that any person who has seen both will have no hesitation in locating the disease: and if the network, curved filaments, and globules, appear to others as they do unto me, the various muscæ will be ascribed to affections of the structure which they resemble.

*Med. Gazette. October* 20, 1838.

